# Association between atherosclerosis and height loss among older individuals

**DOI:** 10.1038/s41598-024-57620-y

**Published:** 2024-04-02

**Authors:** Yuji Shimizu, Kazuhiko Arima, Hirotomo Yamanashi, Shin-Ya Kawashiri, Yuko Noguchi, Yukiko Honda, Seiko Nakamichi, Yasuhiro Nagata, Takahiro Maeda

**Affiliations:** 1 Department of General Medicine, Nagasaki University Graduate School of Biomedical Sciences, Nagasaki, 852-8501 Japan; 2 Epidemiology Section, Division of Public Health, Osaka Institute of Public Health, Osaka, 537-0025 Japan; 3 Department of Public Health, Nagasaki University Graduate School of Biomedical Sciences, Nagasaki, 852-8523 Japan; 4 Leading Medical Research Core Unit, Nagasaki University Graduate School of Biomedical Sciences, Nagasaki, 852-8523 Japan; 5 Department of Community Medicine, Nagasaki University Graduate School of Biomedical Sciences, Nagasaki, 852-8523 Japan; 6 Department of Social Epidemiology, Graduate School of Medicine and School of Public Health, Kyoto University, Kyoto, 606-8315 Japan; 7 Nagasaki University Health Center, Nagasaki, 852-8521 Japan

**Keywords:** Risk factors, Cardiovascular diseases, Biomarkers, Predictive markers, Geriatrics

## Abstract

Atherosclerosis and height loss are each reportedly associated with cardiovascular disease. However, no studies have found an association between atherosclerosis and height loss. A retrospective study of 2435 individuals aged 60–89 years who underwent annual health check-ups was conducted. Atherosclerosis was defined as carotid intima-media thickness (CIMT) ≥ 1.1 mm. Height loss was defined as being in the highest quintile of height decrease per year, as in our previous studies. Among study participants, 555 were diagnosed as having atherosclerosis. Independent of known cardiovascular risk factors, atherosclerosis was positively associated with height loss. The adjusted odds ratio (OR) was 1.46 (95% confidence interval, 1.15, 1.83). Essentially the same associations were observed for men and women. The adjusted OR (95% CI) was 1.43 (1.01, 2.04) for men and 1.46 (1.07, 1.99) for women. Among older individuals, atherosclerosis is associated with height loss. This result can help clarify the mechanism underlying the association between height loss and cardiovascular disease.

## Introduction

Height loss is positively associated with death due to cardiovascular disease^1^. A study of older men reported that height loss is independently associated with an increased risk of all-cause mortality and coronary heart disease^2^. Therefore, cardiovascular risk could be associated with height loss among older individuals.

Carotid intima-media thickness (CIMT), a widely used surrogate marker of atherosclerosis^3^, is reported to be positively associated with cardiovascular disease^4^.

Previously, levels of circulating CD34-positive cells, which play an important role in vascular repair^5–7^, were revealed to be inversely associated with height loss among men aged 60–69 years^8^. Since circulating CD34-positive cell count is inversely associated with all-cause and cardiovascular mortality^9,10^, insufficient endothelial repair related to lower CD34-positive cell count might cause height loss among older individuals.

However, CD34-positive cells also play an important role in the development of atherosclerosis; they differentiate into mature cells such as foam cells and macrophages^11^. Since macrophages^12^ and foam cells^13^ contribute to the development of pathological atherosclerosis, CD34-positive cells are necessary for the development of atherosclerosis as evaluated with CIMT^14^.

Such studies raise the question of whether height loss could be associated with lower circulating CD34-positive cell count^8^. Although height loss is reported to be associated with cardiovascular disease^1,2^ and atherosclerosis as evaluated with CIMT is a known risk factor for cardiovascular disease^4^, circulating CD34-positive cells are a known cause of atherosclerosis. Clarifying the association between atherosclerosis as evaluated with CIMT and height loss could help clarify the mechanism of height loss among older individuals. To investigate the association between atherosclerosis and height loss among older individuals, a prospective study of older Japanese individuals aged 60–89 years who underwent annual health check-ups was conducted.

## Material and methods

The methods used in the present study, including CIMT measurement, have been described elsewhere^15^.

### Study population

The study population comprised 2947 residents of Goto city aged 60–89 years who underwent an annual medical check-up during 2014–2016, which was considered the baseline evaluation. Goto city, which is located in western Japan, is surrounded by rural communities. Participants without data on blood pressure (n = 2) or drinking and smoking (n = 7) were excluded. We also excluded 501 participants who did not undergo an annual health check-up during the follow-up period (2015–2019). To avoid the influence of metastatic fractures, participants with an annual decrease in height > 5 cm per year (n = 2) were also excluded. The remaining 2,435 individuals, aged 72.3 years ± 7.0 years (range 60–89 years), were enrolled in the study. The mean follow-up period was 3.3 ± 1.2 years. There were no significant differences in the characteristics of individuals who were included or excluded.

To ensure that participants understood the objective of the study, written consent forms in Japanese were made available. Informed consent was obtained from all study participants. All study procedures were in accordance with the ethical standards of the institutional research committee and the 1964 Declaration of Helsinki and its later amendments. Ethics approval was obtained from the Ethics Committee for Human Research of Nagasaki University. The study was also approved by the Ethics Committee of the Nagasaki University Graduate School of Biomedical Sciences (project registration number: 14051404-15).

### Data collection and laboratory measurements

Trained interviewers obtained information on clinical characteristics. An automatic body composition analyzer (BF-220; Tanita, Tokyo, Japan) was used to calculate body mass index (BMI, kg/m^2^) after measuring height and weight. Blood pressure in the right arm was measured after at least 5 min of rest in a sitting position with a blood pressure measuring device (HEM-907; Omron, Kyoto, Japan) and recorded by a trained observer.

Fasting blood samples were collected in EDTA-2K and siliconized tubes. Serum high-density lipoprotein cholesterol (HDLc), serum triglycerides (TG), hemoglobin A1c (HbA_1C_), and serum creatinine were measured using standard laboratory procedures at SRL, Inc. (Tokyo, Japan). The glomerular filtration rate (GFR) was estimated using an established method recently proposed by a working group of the Japanese Chronic Kidney Disease Initiative^16^.

CIMT was measured with ultrasonography of the left and right common carotid arteries by an experienced vascular technician using LOGIQ Book XP with a 10-MHz transducer (GE Healthcare, Milwaukee, WI, USA). The maximum CIMT values for the left and right common carotid arteries were calculated using digital edge-detection software (Intimascope; MediaCross, Tokyo, Japan) and a previously described protocol^17^. Intimascope is a software developed to minimize measurement errors in CIMT measurement. This software makes it possible to automatically recognize the edges of the internal and external membranes of blood vessels and automatically determine distances at a sub-pixel level (estimated to be 0.01 mm) using a polynomial measurement formula^18^. Baseline subclinical atherosclerosis was diagnosed as CIMT ≥ 1.1 mm, as in previous studies^14,19^ because < 1.1 mm was reported as normal CIMT in a previous study^20^.

Height loss was defined as being in the sex-specific highest quintile of height decrease per year (2.004 mm/year for men and 2.416 mm/year for women), as in our previous studies^21,22^.

### Statistical analysis

The characteristics of the study population are presented as means ± standard deviation (SD) or n (%), except for TG. Since TG values had a skewed distribution, logarithmic transformation was performed and the median (interquartile range) was presented.

Logistic regression was used to calculate odds ratios (ORs) and 95% confidence intervals (CIs) to determine the association between atherosclerosis and height loss. Three different models were used to calculate ORs and 95% CIs. The first model only included adjustment for age and sex (Model 1). Model 2 also included further adjustment for height at baseline. Finally, Model 3 further adjusted for other potential confounding factors: systolic blood pressure (mmHg), BMI (kg/m^2^), drinking status (non-drinker, often drinker, or daily drinker), smoking status (never smoker, former smoker, or current smoker), HDLc (mg/dL), TG (mg/dL), HbA1c (%), and GFR (mL/min/1.73 m^2^). We also performed sex-specific analyses.

All statistical analyses were performed with SAS for Windows version 9.4(SAS Inc., Cary, NC). *P*-values < 0.05 were regarded as statistically significant.

## Results

### Characteristics of the study population in relation to atherosclerosis

Among the present study population, 555 participants were diagnosed as having atherosclerosis at baseline. Table 1 shows the clinical characteristics of the study population by atherosclerosis status at baseline. Compared with participants without atherosclerosis, there was a significantly higher prevalence of men among participants with atherosclerosis. Participants with atherosclerosis were significantly older and had higher BMI, systolic blood pressure, and HbA1c and significantly lower diastolic blood pressure, HDLc, and GFR.

**Table 1:** Characteristics of study population.

	Atherosclerosis		*p*
	Absent	Present	
No. of participants	1880	555	
Men, %	35.4	48.6	< 0.001
Age	71.5 ± 6.9	74.7 ± 6.9	< 0.001
Body mass index (BMI), kg/m^2^	23.1 ± 3.3	23.4 ± 3.1	0.021
Systolic blood pressure, mmHg	133 ± 16	137 ± 18	< 0.001
Diastolic blood pressure, mmHg	77 ± 11	75 ± 12	< 0.001
Daily drinker, %	16.6	13.7	0.101
Current smoker, %	7.2	7.8	0.652
Triglycerides, mg/dL	91 [67, 127]*^1^	92 [67, 125]*^1^	0.906*^2^
HDL-cholesterol, mg/dL	60 ± 15	57 ± 14	< 0.001
Glycated hemoglobin (HbA1c), %	5.7 ± 0.5	5.8 ± 0.6	0.007
GFR, mL/min/1.73m^2^	69.1 ± 14.1	66.7 ± 15.2	< 0.001
Height at baseline, cm	154.6 ± 8.5	155.4 ± 8.8	0.081

Values were mean ± standard deviation.

GFR, glomerular filtration rate.

*1: Values are median [the first quartile, third quartile].

*2: Logarithmic transformation was used.

### Association between atherosclerosis and height loss

Table 2 shows the ORs and 95% CIs for height loss by atherosclerosis status among all participants. Atherosclerosis was significantly positively associated with height loss. Even after adjustment for height at baseline (Model 2) and known cardiovascular risk factors (Model 3), this association remained significant.

**Table 2:** Odds ratios (95% confidence intervals) for height loss by atherosclerosis status.

	Atherosclerosis		*p*	*p* (Goodness of fit test)
	Absent	Present		
Total				
No of participants	1880	555		
No with height loss (%)	332 (17.7)	154 (27.7)		
Model 1	Reference	1.49 (1.18, 1.87)	< 0.001	0.706
Model 2	Reference	1.48 (1.09, 2.01)	< 0.001	0.690
Model 3	Reference	1.46 (1.15, 1.83)	0.002	0.596

Model 1: adjusted for sex and age. Model 2: adjusted for sex, age, + height at baseline. Model 3: adjusted for sex, age, height, + systolic blood pressure, body mass index, smoking status, drinking status, triglycerides, high density lipoprotein-cholesterol, hemoglobin A1c, renal function (estimated glomerular filtration rate).

Table 3 shows sex-specific associations between atherosclerosis and height loss. Independent of known confounding factors, atherosclerosis was positively associated with height loss among both men and women.

**Table 3:** Sex-specific odds ratios (95% confidence intervals) for height loss by atherosclerosis status.

	Atherosclerosis		*p*	*p* (Goodness of fit test)
	Absent	Present		
Men				
No of participants	666	270		
No with height loss (%)	117 (17.4)	70 (25.9)		
Model 1	Reference	1.49 (1.04, 2.08)	0.028	0.132
Model 2	Reference	1.48 (1.05, 2.09)	0.027	0.926
Model 3	Reference	1.43 (1.01, 2.04)	0.045	0.051
Women				
No of participants	1214	285		
No with height loss (%)	215 (17.7)	84 (29.5)		
Model 1	Reference	1.49 (1.09, 2.02)	0.012	0.961
Model 2	Reference	1.48 (1.09, 2.01)	0.013	0.690
Model 3	Reference	1.46 (1.07, 1.99)	0.017	0.329

Model 1: adjusted only for age. Model 2: adjusted for age and height at baseline. Model 3: adjusted for age, height, + systolic blood pressure, body mass index, smoking status, drinking status, triglycerides, high density lipoprotein-cholesterol, hemoglobin A1c, and renal function (estimated glomerular filtration rate).

### Sensitivity analysis

To assess sensitivity, we performed the main analyses with height loss defined as being in the highest quartile of height decrease per year, as in our previous study^21,22^. We obtained essentially the same results. The OR (95% CI) for height loss and atherosclerosis was 1.34 (1.08, 1.67) for Model 1, 1.34 (1.08, 1.67) for Model 2, and 1.31 (1.05, 1.62) for Model 3.

## Discussion

The main finding of the present prospective study is that among older participants, atherosclerosis is positively associated with height loss. This association was observed among both men and women.

A previous retrospective study of Japanese individuals aged 40–74 years revealed an independent positive association between hypertension and height loss among men^23^. Hypertension, which is a well-known cardiovascular risk factor in Asia^24^, is closely associated with atherosclerosis as evaluated with CIMT^25^. CIMT, a widely used surrogate marker of atherosclerosis^3^, is positively associated with cardiovascular disease^4^. Therefore, higher cardiovascular risk should be associated with height loss among older individuals. In addition, atherosclerosis as evaluated with CIMT could be positively associated with height loss among older individuals.

Early endothelial dysfunction predicts progression of CIMT^26^. Because progression of CIMT is a result of aggressive endothelial repair^27^, endothelial dysfunction might be underlying the process of height loss.

Insufficient endothelial repair also causes endothelial dysfunction. Since CD34-positive cells play an important role in vascular repair^5–7^, a shortage of CD34-positive cells might lead to insufficient endothelial repair^28^. Circulating CD34-positive cell count, which has been reported to be inversely associated with all-cause and cardiovascular mortality^9,10^, has also been reported to be inversely associated with height loss^8^.

However, active arterial wall thickening (CIMT increase ≥ 0.01 mm/year) requires CD34-positive cells^14^. Thus, characteristics associated with a lower chance of CIMT progression being linked with a higher risk of height loss seems like a contradiction. This contradiction might be explained by the reduction in circulating CD34-positive cells due to consumption.

The development of atherosclerosis (CIMT ≥ 1.1 mm), which is the result of aggressive endothelial repair, is inversely associated with active arterial wall thickening^14,19^. Since aggressive endothelial repair induces a shortage of CD34-positive cells due to consumption, participants with established atherosclerosis (CIMT ≥ 1.1 mm) could have lower levels of circulating CD34-positive cells.

Therefore, although circulating CD34-positive cell count is inversely associated with height loss^8^, established atherosclerosis could be positively associated with height loss among older individuals. However, the pathological mechanism underlying the association between atherosclerosis and height loss has not yet been clarified.

Intervertebral disc degeneration and compression vertebrae fractures related to osteoporosis are known causes of height loss among older individuals. Disc degeneration is associated with atherosclerosis of the abdominal aorta^29,30^. Oxidative stress activates intervertebral disc degeneration^31^, progression of osteoporosis^32^, and development of atherosclerosis^33^. Since CIMT is associated with calcification of the abdominal aorta^34^, oxidative stress might be underlying the association between height loss and atherosclerosis as evaluated with CIMT.

However, in the present study, men and women had essentially the same associations between height loss and atherosclerosis. Since the prevalence of osteoporosis is much higher among women than among men^35^, the influence of compression vertebrae fractures related to osteoporosis on height loss might be limited.

Anemia is frequently diagnosed in older individuals and it is multifactorial^36^. Clonal hematopoiesis of indeterminate potential (CHIP) is an important explainable factor that induces anemia among elderly individuals. CHIP, which is common in the normal aging population^37^, is associated with an increased risk of anemia based on hemoglobin level^38^. CHIP is also associated with a pro-inflammatory state that has been linked to atherosclerosis^39^ and arteriosclerotic disease^40^. Chronic inflammation induces the progression of atherosclerosis^41^. Aging is a process that reduces hematopoiesis^42^ and increases inflammation^43^. In addition, there is a reduction in the number of circulating hematopoietic stem cells known as CD34-positive cells in elderly individuals as compared to younger individuals^44^. Since there is an inverse association between hemoglobin level and height loss^21^ and between circulating CD34-positive cell count and height loss^8^, the process of aging might have a strong influence on the association between atherosclerosis and height loss.

Furthermore, aging is known to increase oxidative stress^45^. Low CD34-positive cell production related to aging amplifies the association between oxidative stress and hypertension^25^. Hypertension is an independent risk factor for height loss^23^. Therefore, such associations also explain why aging should have a strong influence on the association between atherosclerosis and height loss. In fact, participants with height loss were significantly older than participants without height loss, as shown in our previous study^46^.

One clinical implication of the present study is that atherosclerosis as evaluated with CIMT could be an efficient tool for estimating the risk of height loss among older individuals. Since height loss among older men might increase the risk of all-cause mortality and coronary heart disease^2^, the present findings also help clarify the potential mechanism underlying the association between height loss and cardiovascular disease among older individuals.

The potential limitations of this study warrant consideration. Vertebral fractures associated with osteoporosis and intervertebral disc degeneration might play an important role in height loss among adults, but those data were not available to us, as in our previous studies^8,21–23^. Further investigation with data on those diseases is necessary. An efficient cutoff point to define height loss has not been established. In the present study, we defined height loss as being in the highest quintile of height decrease per year. However, our sensitivity analysis based on quartiles of height decrease per year showed essentially the same associations. Oxidative stress and hypoxia might play important roles in the present associations. However, we had no data to evaluate oxidative stress and hypoxia. Further epidemiological investigations with data on levels of hypoxia inducing factor, superoxide dismutase, and 8-hydroxydeoxyguanosine are required. Diurnal changes in height^47^ might influence the present results. However, CIMT does not have diurnal changes. Thus, diurnal changes in height might weaken the present results even though we found a significant positive association between atherosclerosis and height loss. Since height loss starting in middle age is an independent risk factor for cardiovascular mortality in old age^1^, there is a possibility that the participants included in the analysis represent a healthy survivor cohort. This type of selection bias might weaken the association between height loss and atherosclerosis because higher CIMT is an independent risk factor for cardiovascular disease^4^. However, significant associations between atherosclerosis and height loss were observed in the present study.

## Conclusion

In conclusion, among older Japanese individuals, atherosclerosis as evaluated with CIMT was positively associated with height loss. Although further investigation is necessary, the present findings are helpful for estimating the risk of height loss and clarifying the mechanism that might be underlying the association between height loss and cardiovascular disease.

## Data Availability

According to ethical guidelines in Japan, we cannot provide individual data due to participant privacy considerations. In addition, the informed consent obtained does not include a provision for publicly sharing data. Qualified researchers may apply to access a minimal dataset by contacting Prof. Takahiro Maeda, Principal Investigator, Department of General Medicine, Nagasaki University, Nagasaki, Japan at tamaeda@nagasaki-u.ac.jp or the Office of Data Management at ritouken@vc.fctv-net.jp. Information about data requests is also available online at: https://www.mh.nagasaki-u.ac.jp/soshin/ (accessed on 25th August 2023) and http://www.med.nagasaki-u.ac.jp/cm/ (accessed on 28th August 2023).
